# Supplementary material for: DSP-Related Cardiomyopathy as a Distinct Clinical Entity? Emerging Evidence from an Italian Cohort
Source: Int J Mol Sci. 2023 Jan 27;24(3):2490. doi: 10.3390/ijms24032490 (PMC9916412; doi:10.3390/ijms24032490)
Supplement: Supplementary file 1 [file ijms-24-02490-s001.zip › ijms-2189574-supplementary.pdf]

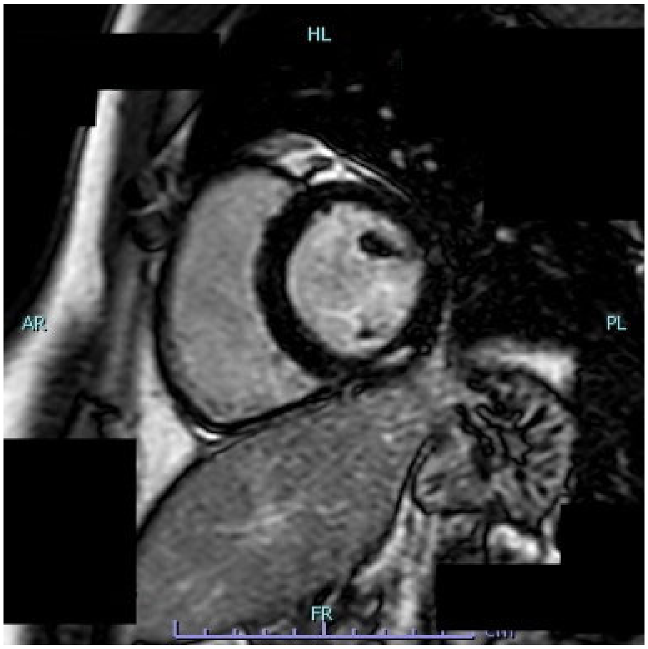

.

Supplementary Figure S1A: Cardiac magnetic resonance imaging of Patient ID 7. Short- axis view. PSIR images shows the presence of subepicardial and midwall LGE in the inferior septum, inferior and posterior wall.

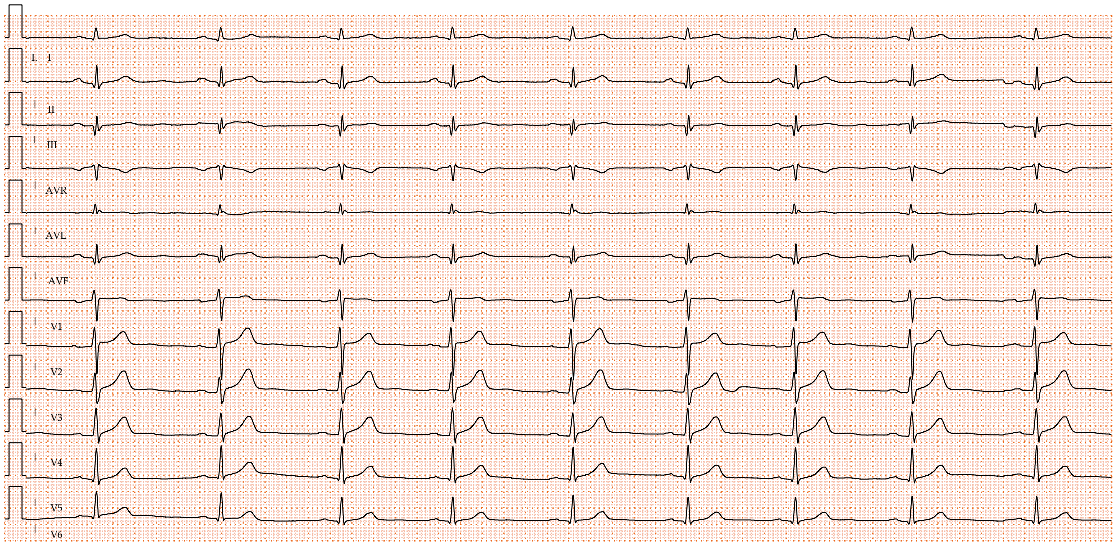

Supplementary Figure S1B: 12-ECG leads of Patient ID 7 shows the presence of pathological Q wave in inferior leads, epsilon-like wave in AVL and low QRS voltages in limb leads.

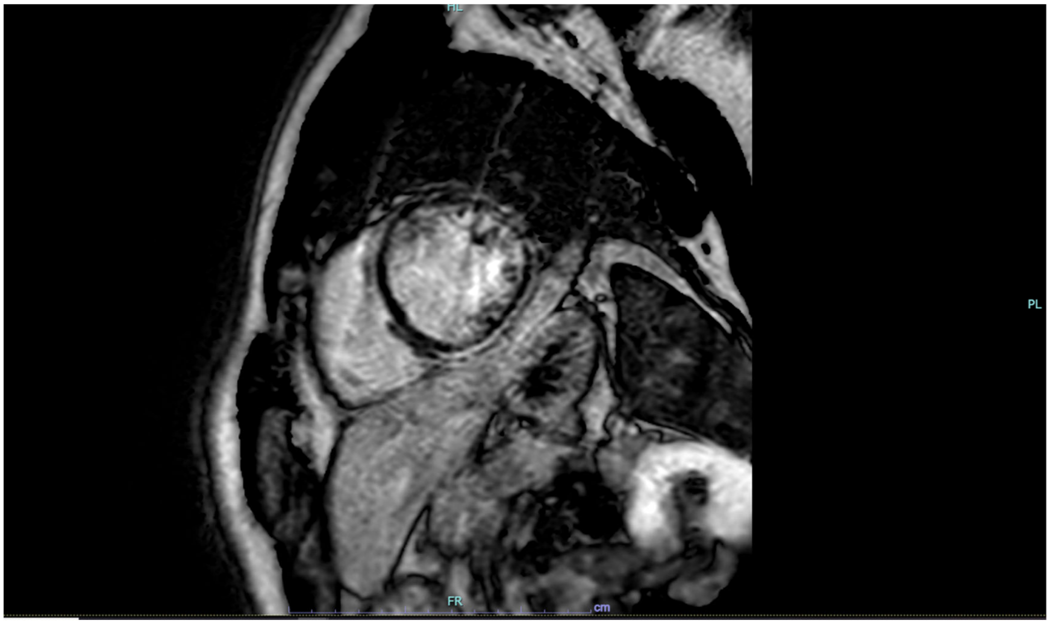

Supplementary Figure S2A: Cardiac magnetic resonance imaging of Patient ID 8. Short- axis view. Ring like pattern LGE involves inferior septum, inferior, posterior and lateral wall.

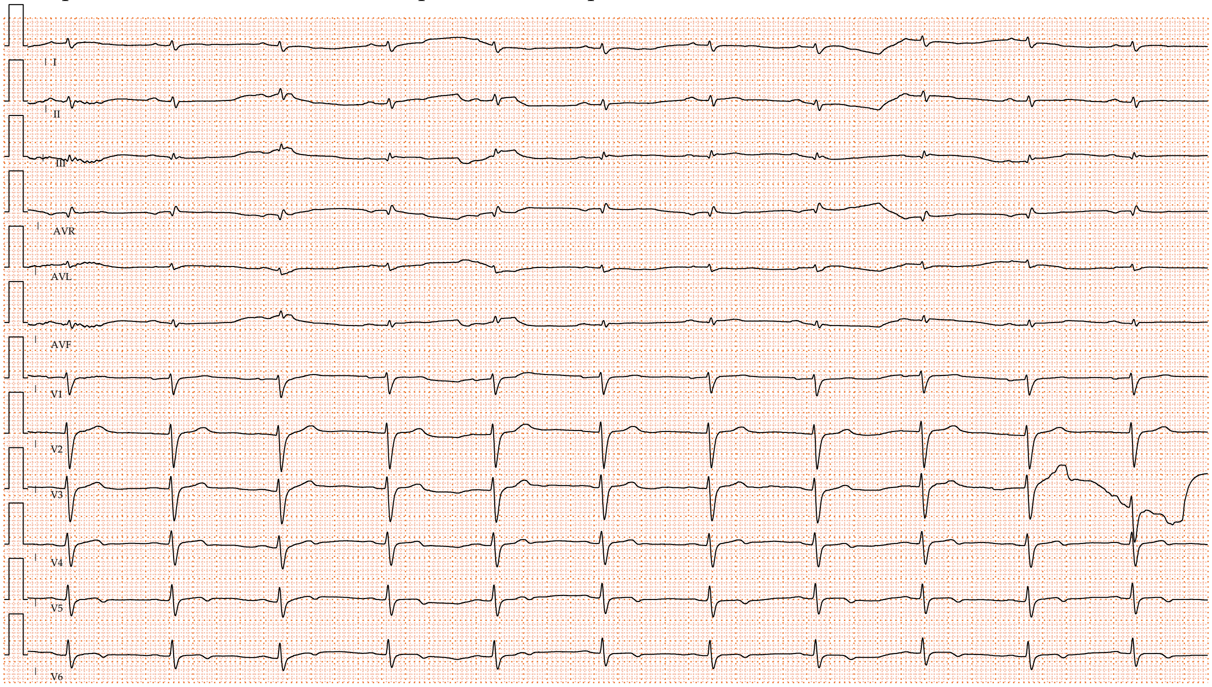

Supplementary Figure S2B: 12-ECG leads of Patient ID 8 shows the presence of low QRS voltages in limb leads and T waves inversion in V5-V6.

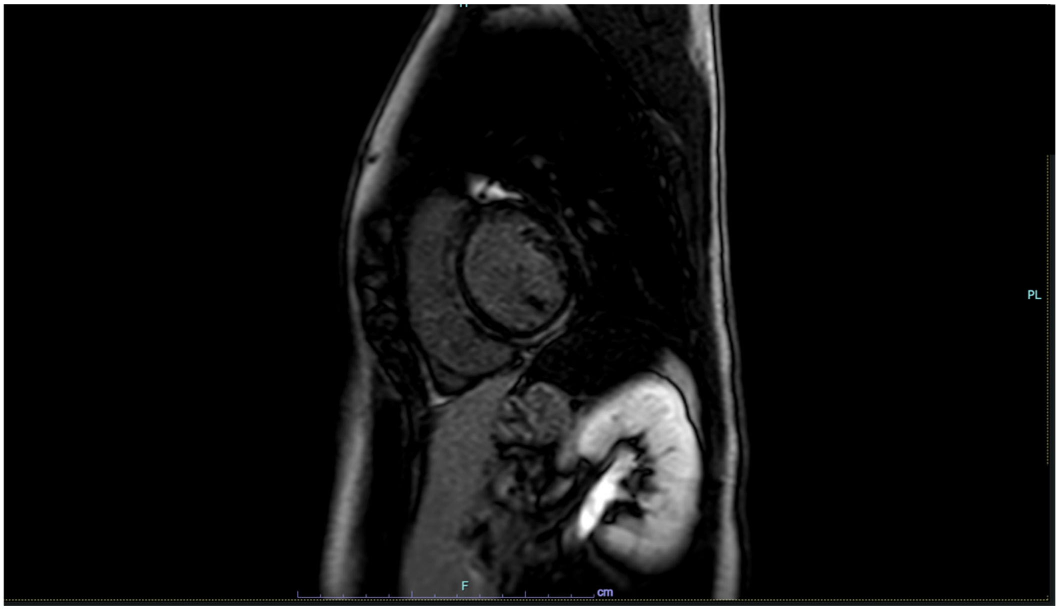

Supplementary Figure S3A: Cardiac magnetic resonance imaging of Patient ID 9. Short- axis view. PSIR images shows ring like LGE pattern involving anterior, septal and inferior wall.

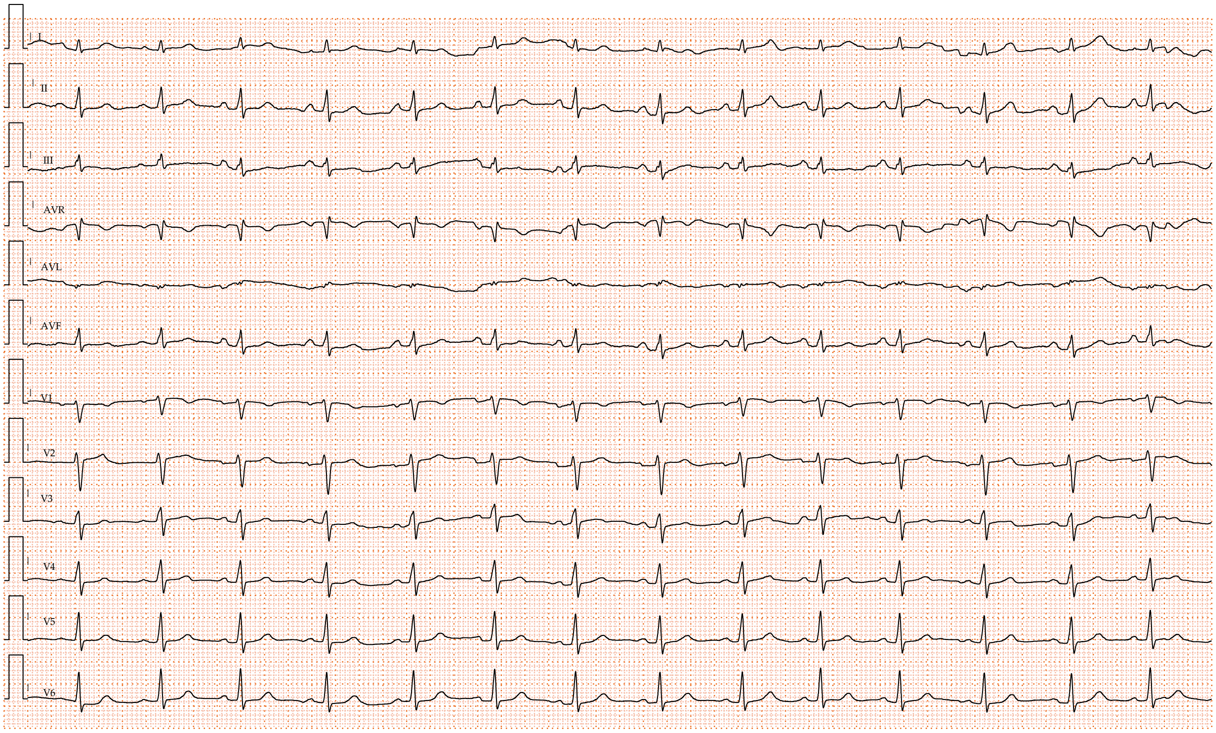

Supplementary Figure S3B: 12-ECG leads of Patient ID 9 shows low QRS voltages (<0.5 mV, peak to peak) in DI, aVL in absence of other repolarization and depolarization ECG abnormalities.

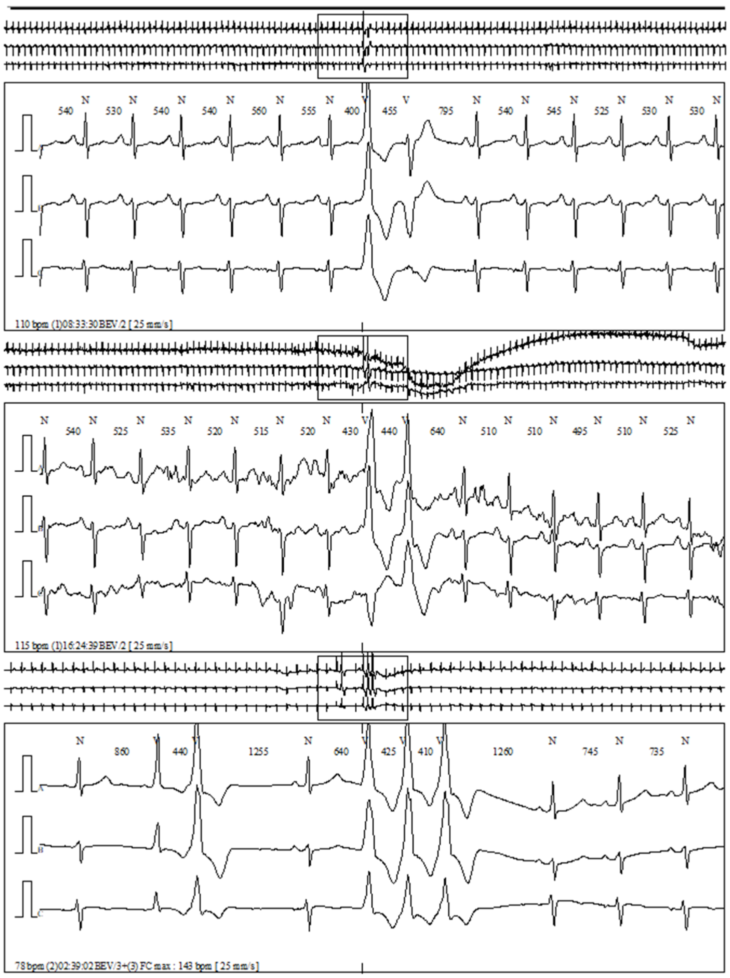

Supplementary Figure S3C: premature ventricular beats in couplets and monomorphic triplets in 24 hours ECG monitoring of Patient ID 9.

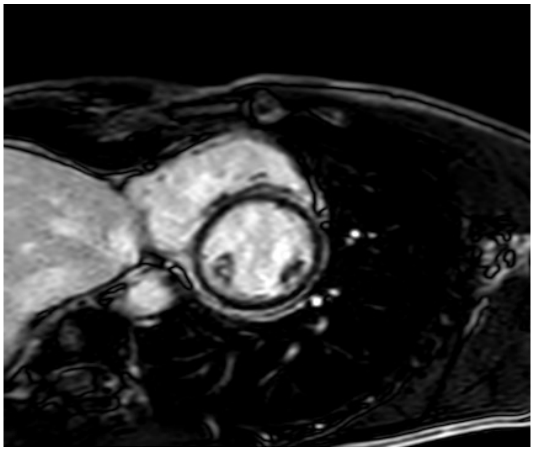

Supplementary Figure S4: Cardiac magnetic resonance imaging of Patient ID 10: Short- axis view. PSIR images shows LV subepicardial ring like LGE pattern.

Table S1: Genes included in NGS target panel

Gene	RefSeq
ACTC1	NM_005159
CACNA1C	NM_001129841
CACNA2D1	NM_000722
CACNB2	NM_201596
CAV3	NM_001234
DSC2	NM_024422
DSG2	NM_001943
DSP	NM_004415
FLNC	NM_001458
GLA	NM_000169
JUP	NM_002230
KCNH2	NM_000238
KCNQ1	NM_000218
LAMP2	NM_013995
LDB3	NM_001171610
LMNA	NM_170707
MYBPC3	NM_000256
MYH7	NM_000257
MYL2	NM_000432
MYL3	NM_000258
PKP2	NM_004572
PLN	NM_002667
PRKAG2	NM_016203
RBM20	NM_001134363
RYR2	NM_001035
SCN5A	NM_00109940
TNNC1	NM_003280
TNNI3	NM_000363
TNNT2	NM_001276347
TPM1	NM_001018006
TTN	NM_001267550
TTR	NM_000371

Table S2: Oligonucleotide sequences used for Sanger sequencing

HGVSc	Exon	Primers
c.6154C>T	24	**Fw:**TGACACCTCCAAGCTG<br>**Rv:**AAGGACATGCTTCTCTTC
c.2497C>T	18	**Fw:**GGAACAAATACTGGGAAATGC<br>**Rv:**TTCTTCAGTGGTTCCCTTCAA
c.2848del	20	**Fw:**GGATAGGCCCAGAAAACAGG<br>**Rv:**GCACCTACCACCAGTCAAAG
c. 1352G>C	11	**Fw:**CTGCATAGCTTCTGGAGAGTG<br>**Rv:**TCAAAGACATTGACTGTGGTGAT
c.5851C>T	24	**Fw:**GCTCACAGTGTATCCAGGGA<br>**Rv:**TGTCGACAGTCAGCTTCTCA
c.3203_3204del	23	**Fw:**ATATGAGCAGCTGGTGCAA<br>**Rv:**TCTCCTTGATGGCTTTCTC
c.5210del	23	**Fw:** AGCCATCAAAATCACCAACC<br>**Rv:** TTTGAGAAACAGTGAAGCTGT
c.170+2T>G	-	**Fw:** GGTAGCGAGCAGCGACCTC<br>**Rv:** GCGGGAGGGGAGAAAGGTA
c.3206_3207del	23	**Fw:**ATATGAGCAGCTGGTGCAA<br>**Rv:**TCTCCTTGATGGCTTTCTC
c.2506C>T	18	**Fw:**GGAACAAATACTGGGAAATGC<br>**Rv:**TTCTTCAGTGGTTCCCTTCAA
c.879_890dup	7	**Fw:**GCCTTTGAACCTCCTGTGCA<br>**Rv:** GAGGGCAACAACACACACTG
c.3724_3739del	23	**Fw:**AGGAGAACCTTGGTTGGCAG<br>**Rv:**CTGAATGGTCTTGGCAGCCT
c.5601G>A	24	**Fw:**GCTCACAGTGTATCCAGGGA<br>**Rv:**TGTCGACAGTCAGCTTCTCA
c.1067C>A	9	**Fw:**GGTGAGAAATTCTCTTTCCAAC<br>**Rv:**TCAGTGCTTTATAAAACACACTC
c.3932_3936del	23	**Fw:**AGGAGAACCTTGGTTGGCAG<br>**Rv:**CTGAATGGTCTTGGCAGCCT
c.816_817delinsAT	7	**Fw:**GCCTTTGAACCTCCTGTGCA<br>**Rv:** GAGGGCAACAACACACACTG
